# Clinical and course indicators of bipolar disorder type I with and without opioid dependence

**Published:** 2010

**Authors:** Amir Shabani, Atefeh Ghanbari Jolfaei, Hajar Ahmadi Vazmalaei, Azizeh Afkham Ebrahimi, Morteza Naserbakht

**Affiliations:** a Bipolar Disorders Research Group, Mental Health Research Centre, Iran University of Medical Sciences and Health Services, Tehran, Iran; b Iran Hospital of Psychiatry, Iran University of Medical Sciences and Health Services, Tehran, Iran; c Tehran Psychiatric Institute, Iran University of Medical Sciences and Health Services, Tehran, Iran

**Keywords:** Bipolar Disorder, Substance Dependence, Substance Abuse, Opioid, Outcome

## Abstract

**BACKGROUND:**

The existing evidence about the clinical situations of the bipolar patients with opioid dependence is scarce. The present study was carried out to compare the clinical features and course of the bipolar disorder type I regarding the two subgroups of opioid dependent and non-dependent.

**METHODS:**

There were 178 adult patients with bipolar disorder type I consecutively referred to the Iran Hospital of Psychiatry, Tehran, Iran, from January 2008 to January 2009 who enrolled in the study. The Persian Structured Clinical Interview for DSM-IV axis I disorders (SCID-I), HDRS-17, and Y-MRS were administered for all patients. Other clinical information was gathered through the face-to-face interviews with the probands and the hospital records. The T test, Chi square test and logistic regression were used to analyze the data.

**RESULTS:**

The mean age of probands were 33.6 ± 11.1 years old and they were mostly male. Among the evaluated indices, the factors gender, anxiety disorders comorbidity, non-adherence, and positive family history were different significantly and independently from the other studied factors between opioid dependent and non-dependent bipolar patients.

**CONCLUSIONS:**

Despite some differences, the opioid dependent and non-dependent bipolar patients did not have any significant difference regarding most of the examined clinical and course indices.

The substance abuse prevalence among patients with bipolar disorder (BD) has been between 17 and 65 percent.^1^ Based on the several studies the alcohol and cannabis have been estimated to be the most common abused substances by the BD patients.^2^ The cocaine and then opioids have stood on the next ranks.^3^ Nevertheless, a multi-center study in Iran showed that the opioid use disorder (OUD) is the most prevalent type of the sub-stance use disorders (SUDs) in the clinical psychiatric patients referred to the psychiatric hospitals.^4^

According to the various studies, the SUD comorbidity in patients with BD is in association with particular features; e.g. earlier onset of BD, higher frequency of rapid cycling, and dysphoric and mixed states, higher rate of psychiatric hospitalization, slower improvement of manic episode,^5^ more suicidal attempts,^5^–^7^ higher rate of personality disorders comorbidity and increased level of social dysfunctioning,^8^ more possibility of chronicity, disability, and mortality, more number of clinical symptoms and relapse, delayed remission, reduced time of relapsing,^9^ poorer therapeutic outcome,^5^,^10^ lack of response to lithium,^5^ lower level of quality of life,^11^ and higher frequency of substance dependence than substance abuse.^12^ Nevertheless, the current knowledge is predominantly based on assessing the patients abusing alcohol or a mixed sample of individuals abusing various drugs, so that the patients with the use disorder of each certain substance remain to be settled. The significance of this issue is particularly drawn from this finding that some clinical indices are related to the type of abused substance in patients with the comorbidity of BD and SUD. For example, Dalton et el^13^ showed that the substance use disorder, but not the alcohol use disorder (AUD), was associated with the attempted suicide rate in patients with BD. On the other hand, the mood stabilizing effects of the opioid drugs have been received attention specifically.^2^ Considering the mentioned issues and also this point that the opioids use prevalence in the Iranian in general^14^ and clinical^4^ populations have been higher in comparison with the western ones, the present study was carried out to compare the clinical features and course of the bipolar disorder type I regarding the two subgroups of opioid dependent and non-dependent.

## Methods

### 

#### Participants

A total of 178 inpatients with bipolar disorder type I consecutively referred to the Iran Hospital of Psychiatry, Tehran, Iran, from January 2008 to January 2009 were enrolled in the study.

The patients who aged 18-65, were diagnosed as BD type I at least since two years ago, gave informed written consent, were able to take part in the interview, and had no mental retardation and history of substance abuse (with the exception of opioids, nicotine and caffeine) entered the study. Finally samples were consisted of 89 patients with and the same number without opioid dependence. Actually, the recruitment of the patients continued until the number of each subgroup participants reached the number 89.

The project was approved by the Research Committee of the Department of Psychiatry, Iran University of Medical Sciences and Health Services.

#### Procedure

The Persian Structured Clinical Interview for DSM-IV axis I disorders (SCID-I),^15^ HDRS-17,^16^ and Y-MRS^17^,^18^ were administered for all pa-tients. The SCID-I is a diagnostic tool which can be administered by a clinician. Its Persian version has had acceptable reliability, validity and feasibility on a large sample of Iranian patients.^15^ The HDRS-17^16^ and Y-MRS^17^,^18^ are the common and useful instruments to measure the severity of the mood symptoms. Also, demographic features, hospitalization rate, number of the months with non-compliance in the last year, age at the first major mood episode and at the first hospitalization, and family history (first-degree) for the major psychiatric disorders including psychotic, mood, anxiety and substance use disorders were identified.

Two trained last-year residents of psychiatry (from Iran University of Medical Sciences and Health Services) did the face-to-face interviews with the probands. The patients were interviewed at the last days being in the hospital. The hospital records were another resource of the gathered information.

#### Statistical Analysis

The data was analyzed by the Software Package for Social Sciences (SPSS, version 14). Also the T test, Chi square test, and logistic regression were used to analyze the findings.

## Results

The mean age of probands were 33.6 ± 11.1 years old and they were mostly male (77.5%). The demographic features of the opioid dependent and non-dependent patients with BD are seen in table 1. According to this table, gender ratio, and marital and occupational status were significantly different between the two groups.

**Table 1:** Demographic features of the participants with bipolar I disorder (n = 178). Data are mean ± SD or n (%)

Variable		Opium dependent (n = 89)	Non opium dependent (n = 89)	P value
Male		79 (88.7)	59 (66.3)	0.01
Marital status				
	Never married	32 (36.0)	49 (55.1)	0.02
	Married	42 (47.2)	31 (34.8)	
	Divorced	15 (16.9)	9 (10.1)	
Age (years)		33.4 ± 10.8	34.4 ± 11.2	NS*
Education				
	Under diploma	63 (72.4)	53 (59.6)	NS
	Diploma	20 (23.0)	26 (29.2)	
	College	4 (4.6)	10 (11.2)	
Occupation**				
	Unemployed	59 (66.3)	49 (55.1)	0.001
	Employed	24 (27.0)	14 (15.7)	
	Housewife	6 (6.7)	26 (29.2)	

* Non-significant

** There was missing information about two cases

The clinical features of the opioid dependent and non-dependent patients with bipolar disorder type I are presented in table 2. Among these features, three items including anxiety disorders comorbidity, non-adherence to pharmacotherapy, and positive family history of major psychiatric disorders were significantly different between two mentioned groups (p < 0.001).

**Table 2:** Clinical features of the opioid dependent and non-dependent patients with bipolar I disorder (n = 178). Data are mean ± SD or n (%)

Variable		Opioid dependent (n = 89)	Non opioid dependent (n = 89)	P value
Anxiety disorders comorbidity		65 (73.0)	35 (39.3)	0.001
Current mood episode				
	Manic	56 (62.9)	64 (71.9)	
	Major depressive	15 (16.9)	15 (16.9)	NS*
	Mixed	18 (20.2)	10 (11.2)	
Type of index episode				
	Manic	49 (55.1)	65 (73.0)	
	Major depressive	30 (33.7)	19 (21.3)	NS
	Mixed	10 (11.2)	5 (5.6)	
Age at the first hospitalization (years)		24.8 ± 7.1	26.3 ± 8.9	NS
Age at the first major mood episode (years)		23.7 ± 6.5	24.7 ±8.7	NS
Number of hospitalizations		4.1 (2.8)	3.5 (2.4)	NS
No pharmacotherapy in one year (months)		7.8 ± 4.7	5.4 ± 4.3	0.001
HDRS-17 (mean)		7.3 (7.0)	5.7 (4.8)	NS
Y-MRS (mean)		29.6 (12.8)	30 (12.9)	NS
Positive family history**		41 (46.1)	57 (64.8)	0.001

* Non-significant

** There was missing information about one cases

The variables which were different significantly between the opioid dependent and non-dependent groups were analyzed using the logistic regression analysis (Table 3). As seen in this table, each of the factors gender, anxiety disorders comorbidity, non-adherence, and positive family history is different significantly and independently from the other studied factors between opioid dependent and non-dependent bipolar patients. Therefore, other demographic factors which were different between the two groups based on the simple tests (marital and occupational status), did not have any significant difference after logistic regression.

**Table 3:** The associations of different variables with the opioid dependence in patients with bipolar I disorder through a logistic regression analysis

Variable	β	SE	Wald	df	P	EXP B	95.0% C.I. for EXP(B)	
							Lower	Upper
Gender	2.626	0.706	13.840	1	0.000	13.821	3.465	55.137
Marital status	0.220	0.293	0.565	1	0.452	0.802	452	1.425
Occupation status	0.424	0.356	1.415	1	0.234	1.528	760	3.073
Anxiety disorders comorbidity	1.561	0.379	16.948	1	0.000	4.762	2.265	10.011
Non-adherence	0.091	0.043	4.452	1	0.035	1.095	1.006	1.192
Positive family history	0.799	0.368	4.706	1	0.030	2.224	1.080	4.579

## Discussion

This study demonstrated no significant difference between dependent and non-dependent bipolar type I patients considering most of the assessed clinical variables; i.e. type of the index mood episode, type of the current mood episode, age at the first major mood episode and the first hospitalization, number of hospitalizations, and severity of the current mood symptoms. Given that there is a little information about the clinical situation and course of the comorbidity of opioid dependence and BD, comparison of the present findings with the data of the studies on the BD patients with SUD indicates an inconsistency. Based on the previous studies, BD in the patients with SUD has common features such as starting at the lower age, higher severity of clinical symptoms, increased recurrence, slower and poorer response to treatment, more impairment in functioning, and lower level of quality of life. For example, Himmelhoch et al^19^ and Keller et al^20^ showed that these patients experienced more severe manifestations of BD like rapid cycling, dysphoric mania and mixed state. Also, in patients with co-occurrence of BD and AUD, AUD was associated with psychosis within first mood episode which pointed out the high level of severity of the indexed episode.^21^ More severity of BD among patients with SUD could be observed reviewing the studies which have demonstrated more disturbances in social functioning,^8^,^21^ increased disability and mortality,^7^ and more episodic recurrence^22^,^23^ in these patients. Moreover, quality of life-the index has received much atten-tion in recent years-has been measured in the individuals with BD and its decreased level has been reported.^11^ Another important factor with the demonstrated relationship with higher severity and poorer outcome of mood disorder^24^–^27^ is “age at onset”. The low age at onset of BD in patients with SUD has been reported repeatedly^5^,^9^ that could be a more emphasis on the fact that the severity of BD associated with SUD is more than of BD without any SUDs. Finally, decreased response of patients with this dual diagnosis to pharmacotherapy for BD indicates the poorer prognosis of this comorbidity.

Therefore, given that in the present study there were not significant differences between the two groups of probands with and without OUD for most of the evaluated factors, the most important finding of this study might be the existing of a probable difference between the effect of OUD comorbidity and of substance use disorders in general on the clinical situation and course of bipolar disorder. In other word, a specific effect from opioid abuse was seen that could have some implications and propose some hypotheses.

Nevertheless, this study showed a few significant differences between the dependent and non-dependent BD patients: gender, positive family history for psychiatric disorders, anxiety disorders comorbidity and non-adherence. The difference in gender ratio^4^,^14^ was according to the previous evidence highly predictable, but about the difference in the rate of positive family history, the findings in the literature are conflicting^21^,^28^ and difficult to reconcile.

The anxiety disorders are common not only in the patients with BD^29^,^30^ but also in the SUD cases.^31^,^32^ A study by Kolodziej et al proposed an increase in substance use disorders (especially cocaine and amphetamine) frequency among patients with the comorbidity of BD and posttraumatic stress disorder (but not other anxiety disorders).^33^ The present study in line with the previous ones indicates that the rate of anxiety disorders co-occurrence in bipolar patients with SUD is high. Another finding is that the mentioned increment is independent from other contributing evaluated factors and consequently is valid. Hence, to manage the patients with BD and concurrent opioid dependence, the clinicians should probe for any anxiety symptoms and disorders and treat them. Also, regarding the high frequency of co-occurrence of BD, SUD and anxiety disorders, it is suggested that the clinical features, course and treatment of this triad be studied.

The two groups of individuals with and without OUD were different in terms of pharmacotherapy compliance that is consistent with the literature.^10^,^34^,^35^ To explain why the mood stabilizing treatment adherence is decreased in bipolar patients with SUD, some hypotheses are proposed which need to be tested; e.g. the substances are able to subside the mood symptoms^36^ the substances would be able to change patients’ insight to the mental disorder; the cultural beliefs might change the patients’ attitude toward pharmacotherapy; substancedrug interactions may lead to some adverse events resulting in poor compliance; and the specific effects of BD-SUD comorbidity might be a key for reaching the proper answer. Based on the authors’ experience in the Iranian culture, some patients believe in the therapeutic effects of opium. They imagine that they should use either one for going on the therapy: opium or prescribed drug! So, they may state that they have stopped the medication because of starting opium use!

## Conclusions

To sum up, despite the difference in anxiety disorders comorbidity and non-adherence rate between the opioid dependent and non-dependent bipolar patients, the two groups did not have any significant difference for most of the examined clinical and course indices. Hence, some hypotheses are presented about the relationships between opioid use and bipolar disorder which each one needs to be investigated through future studies. However, the present findings should be settled considering the methodological limitations. Given non-random sampling, the sample was not inevitably representative. Furthermore, the substance that the patients were dependent on was simply from opioid group and not alcohol nor other substances concurrently. Thus it should be considered that the findings are just related to opioid drugs. These data were obtained through a cross-sectional and retrospective assessment and a few of them were not gathered based on a standardized instrument. Also, having an anamnestic approach to getting some historical data could be another limitation. Regarding some other important clinical features like quality of life, and doing prospective and longitudinal studies, therapeutic interventions, and biological investigations, like genetic and basic science studies will prepare more evidence on the matter of similarities and dissimilarities of these two groups of patients with bipolar disorder.
